# The global burden of colorectal cancer attributable to high body-mass index in 204 countries and territories: findings from 1990 to 2021 and predictions to 2035

**DOI:** 10.3389/fnut.2024.1473851

**Published:** 2024-11-20

**Authors:** Xiaoqian Jin, Danfeng Dong, Zixuan Xu, Mingming Sun

**Affiliations:** Department of Gastroenterology, Shanghai Tenth People's Hospital, Tongji University School of Medicine, Shanghai, China

**Keywords:** colorectal cancer, high body-mass index, mortality, disability adjusted life years, global burden, sociodemographic index

## Abstract

**Background:**

The association between high body-mass index (BMI) and colorectal cancer (CRC) has been confirmed and gained attention. However, a detailed understanding of the disease burden of high BMI and CRC remains lacking.

**Objective:**

This study aimed to assess the temporal and geographical trends of CRC deaths and disability-adjusted life years (DALYs) caused by high BMI globally from 1990 to 2021, providing effective guidance for developing prevention and treatment strategies.

**Methods:**

We used data from the 2021 Global Burden of Disease study to assess the global, regional, and national Deaths, DALYs, age-standardized mortality rate (ASMR), and age-standardized DALY rates (ASDR) caused by CRC related to high BMI, and further calculated the estimated annual percentage change (EAPC). We also considered factors such as gender, age, and sociodemographic index (SDI). We explore the relationship between EAPC and ASMR/ASDR (1990) and between EAPC and SDI (2021). Further, the autoregressive integrated moving average (ARIMA) model was applied to predict the disease burden from 2022 to 2035. The risk factors were calculated by Population Attributable Fraction (PAF).

**Results:**

In 2021, CRC caused by high BMI resulted in 99,268 deaths (95% Uncertainty Interval (UI): 42,956–157,949) and 2,364,664 DALYs (95% UI: 1,021,594–3,752,340) globally, with ASMR and ASDR being 1.17 per 100,000 population (95% UI: 0.51–1.87) and 27.33 per 100,000 population (95% UI: 11.8–43.37), respectively. The disease burden was higher in males and the elderly, with significant differences between regions and sociodemographic groups. From 1990 to 2021, the ASMR for CRC associated with high BMI revealed little change globally, while the ASDR revealed an upward trend. The burden of CRC caused by high BMI has shifted from high SDI regions to low and low-middle SDI regions. Additionally, from 2022 to 2035, ASMR and ASDR are expected to increase in males, while ASMR and ASDR in females are expected to remain relatively stable.

**Conclusion:**

From 1990 to 2021, the number of deaths and DALYs related to high BMI-associated CRC globally, as well as ASMR and ASDR, continue to rise. We predict that ASMR and ASDR may further increase by 2035, making it crucial to take timely and targeted interventions.

## 1 Introduction

Colorectal cancer (CRC) is a malignant tumor with high incidence and mortality rates worldwide and poses a significant threat to public health. As the most common malignancy of the digestive system, CRC ranks third in cancer incidence and second in mortality worldwide (1, 2). Recently, with population aging, Westernized dietary patterns, and the prevalence of sedentary lifestyles, the incidence of CRC has exhibited a continuous upward trend, specifically in developing countries. The number of CRC cases and deaths worldwide has increased in recent years. Additionally, differences in CRC incidence and mortality rates are associated with varying levels of human development and regional disparities (3). Over the past 30 years, the trend in CRC incidence has remained stable or even declined in high sociodemographic index (SDI) countries, while it has been on the rise in low-income and transitioning economies. This indicates an unequal distribution of the CRC burden worldwide. Over time, this inequality seems to worsen, leading to a growing global gap in the CRC burden. Low- and middle-income countries are bearing an increasing CRC burden (3, 4).

Based on the Global Burden of Disease (GBD) 2021, there are over 20 risk factors contributing to CRC, among which dietary risk factors are the most significant, followed by metabolic risk factors (1, 5). Among the metabolic risk factors, a high body mass index is the most prominent. Numerous research conclusions have already been established regarding CRC attributed to dietary risk factors, which we will not elaborate on here (5). Next, we will primarily explore the epidemiological trends of CRC attributed to high BMI among metabolic risk factors and describe risk factors using PAF.

Body-mass index (BMI) is obtained by dividing a person’s weight (kg) by the square of their height (m). It is a direct indicator used to classify adults into three categories: underweight, BMI below 18.5; overweight, BMI 25.0–29.9; obese, BMI 30.0 and above (6). Numerous studies have demonstrated that a high BMI increases cancer risk, with an elevated BMI being considered an established risk factor for CRC and 12 other cancers. A meta-analysis by the World Cancer Research Fund indicates that for every 5 kg/m^2^ increase in BMI, the risk of CRC increases by 5% (7, 8). According to GBD 2021, 9.51% of the mortality burden and 9.69% of the disability-adjusted life years (DALYs) burden is attributable to high BMI (1, 9).

The mechanisms by which a high BMI affects CRC risk are complex and diverse. First, individuals with high BMI, hyperlipidemia, and insulin resistance have low-grade systemic inflammation, promoting tumor cell proliferation and angiogenesis while reducing apoptosis (10). Insulin resistance and hyperinsulinemia may promote tumorigenesis through the IGF-1 signaling pathway, and adipokines, such as leptin and adiponectin, secreted by adipose tissue may also be involved in the development and progression of CRC (11). Additionally, the mechanisms contributing to CRC may include physical activity levels, dietary patterns, genetic background, and the gut microbiome (12). Despite the extensive evidence provided by existing studies, limitations remain, such as reliance on cross-sectional or retrospective studies, making it difficult to determine causality. BMI, as a measure of obesity, also has some limitations. Future research may need to incorporate other metrics, such as waist circumference and body fat percentage, to comprehensively assess the relationship between body fat and CRC risk (13, 14).

By analyzing data from 204 countries and regions between 1990 and 2021, this study provides a comprehensive assessment of the impact of high BMI on the CRC burden, which is crucial for developing effective public health policies and personalized prevention strategies. Furthermore, we developed a model to forecast the global disease burden of high BMI-related CRC from 2022 to 2035. Future research should further elucidate the molecular mechanisms involved and explore effective interventions to reduce the risk of high BMI-related CRC.

## 2 Materials and methods

### 2.1 Data sources

GBD 2021 provides a comprehensive overview of disabilities and deaths caused by high BMI leading to CRC from 1990 to 2021 across different countries, ages, and genders. It estimated the incidence, prevalence, mortality, years of life lost (YLLs), years lived with disability (YLDs), and DALYs due to 369 diseases and injuries in 204 countries and regions. DALYs are a measure that combines years of YLLs and YLDs (15). We obtained the number of deaths and DALY data caused by high BMI leading to CRC in 204 countries and regions from the Global Health Data Exchange (GHDx) website,^1^ age-standardized mortality rates (ASMR), and age-standardized DALY rates (ASDR) (16, 17). The 204 countries and regions were further divided into 21 regions based on their geographical location. Moreover, data analysis adopted a stratified approach, dividing age into 16 different groups, with 15 being 5-year age intervals (20–94 years, in 5-year intervals) and one being individuals aged 95 years and above.

We separately obtained SDI data for 1990–2021 from the GHDx. SDI is a good indicator of socioeconomic development related to health. It is a composite indicator of lag-distributed income *per capita*, the average educational attainment of the population aged 15 years and above, and the total fertility rate under the age of 25, ranging from 0 to 1. According to the SDI quintiles, 204 countries or regions were divided into five quintiles: low SDI (< 0.45), low-middle SDI (≥ 0.45 and < 0.61), middle SDI (≥ 0.61 and < 0.69), high-middle SDI (≥ 0.69 and < 0.80), and high SDI (≥ 0.80) (18, 19).

### 2.2 Statistical analyses

In this study, we analyzed the global, national burden of CRC caused by high BMI using the ASMR and ASDR (per 100,000 population) to reflect the actual situation of deaths and DALYs (20). To assess trends, we used the linear regression equation (y = *α* + βx + *ε*), where y represents the age-standardized rate (ASR), which refers to ASIR, ASMR, or ASDR and x represents the calendar year. The estimated annual percentage change (EAPC) was calculated as 100 × (exp(*β*) − 1), with its 95% confidence interval (CI) determined using the linear regression model. If the EAPC value and its 95% CI lower limit are both greater than 0, the ASR is considered to exhibit an increasing trend annually. If the EAPC value and its 95% CI upper limit are both less than 0, the ASR is considered to exhibit a decreasing trend annually. If the EAPC is close to 0, the ASR is considered stable (21). This method is widely used to quantify and summarize temporal changes in ASR (22, 23). Statistical analyses were performed using R software (version 4.4.1), with *p*-values <0.05 considered statistically significant.

To analyze the factors influencing the EAPC of the CRC burden related to high BMI, we used Pearson correlation analysis to examine the relationship between EAPC and ASR in 1990 and the relationship between EAPC and SDI scores in 2021, with data analysis performed using R software (version 4.4.1), two-tailed *p* < 0.05 was considered statistically significant.

### 2.3 Autoregressive integrated moving average model

To forecast the global disease burden of high BMI-related CRC from 2022 to 2035, we used an autoregressive integrated moving average (ARIMA) model. It forecasts future values based on past properties (limitations and prediction errors). The ARIMA model is defined by three parameters: p, d, and q, where p is the order of autoregression, q is the order of moving average, and d is the degree of difference. The ARIMA equation is

$$Y_{t}=\alpha +\phi_{1}Y_{t-1}+\phi_{2}Y_{t-2}+\ldots +\phi_{p}Y_{t-p}+\varepsilon_{t}+\phi_{1}\varepsilon_{t-1}+\ldots +\phi_{q}\varepsilon_{t-q'}$$

where *ϕ* and *θ* are the autoregressive and moving average parameters, respectively. *Y_t_* represents the differenced time series, and *ε_t_* is the value of the random shock at time *t*. *α* is a constant (24, 25). After multiple tests, we identified the optimal model using the ARIMA (1,1,2) model to predict ASMR and ASDR for males and the ARIMA (2,0,0) model to predict ASMR and ASDR for females.

## 3 Results

### 3.1 Trends of global burden of CRC from 1990 to 2021

From 1990 to 2021, globally, the incidence of CRC revealed an increasing trend, with the number of cases increasing from 916,584 (95% UI 866,238–951,895) in 1990 to 2,194,143 (95% UI: 2,001,272–2,359,390) in 2021, with an EAPC of 0.15 (95% CI: 0.12–0.19) (Table 1). In 2021, among the quintiles of SDI, areas with high SDI had the highest ASIR, but its annual growth rate decreased (EAPC = −0.25; 95% CI: −0.35 to −0.16), while the region with the highest annual growth rate was the middle SDI (EAPC = 1.38; 95% CI: 1.3–1.46) (Table 1). At the regional level of GBD, the region with the fastest growth in ASIR was Central Latin America (EAPC = 2.05; 95% CI: 1.99–2.11), and the region with the largest annual decline in ASIR was High-income North America (EAPC = −0.8; 95% CI: −0.92 to −0.67) (Table 1).

**Table 1:** Incidence of colorectal cancer in 1990 and 2021 by sex, SDI, and GBD region, with estimated annual percentage change from 1990 to 2021.

Incidence of colorectal cancer in 1990 and 2021 by sex, SDI, and GBD region, with estimated annual percentage change from 1990 to 2021					
Characteristics	Incidence in 1990	ASIR per100,000 in 1990	Incidence in 2021	ASIR per100,000 in 2021	EAPC (1990–2021)
Global	916,584 (866,238 to 951,895)	24.04 (22.54 to 25.01)	2,194,143 (2,001,272 to 2,359,390)	25.61 (23.32 to 27.52)	0.15 (0.12 to 0.19)
Sex					
Male	469,763 (445,308 to 492,302)	27.31 (25.89 to 28.51)	1,263,462 (1,146,499 to 1,400,377)	31.93 (29.04 to 35.26)	0.5 (0.46 to 0.54)
Female	446,821 (414,136 to 472,553)	21.41 (19.75 to 22.62)	930,681 (824,674 to 1,017,652)	20.17 (17.86 to 22.05)	−0.29 (−0.33 to −0.25)
SDI					
Low SDI	16,472 (12,928 to 18,871)	7.32 (5.82 to 8.36)	36,649 (32,707 to 40,883)	7.39 (6.65 to 8.19)	−0.06 (−0.18 to 0.06)
Low-middle SDI	38,286 (33,160 to 43,284)	6.15 (5.36 to 6.94)	119,416 (109,344 to 130,919)	8.19 (7.51 to 8.96)	0.96 (0.93 to 0.99)
Middle SDI	134,432 (121,315 to 148,566)	12.9 (11.65 to 14.16)	526,190 (462,016 to 595,124)	19.55 (17.14 to 22.04)	1.38 (1.3 to 1.46)
High-middle SDI	250,999 (237,456 to 263,213)	25.58 (24.12 to 26.81)	669,660 (598,306 to 746,397)	34 (30.33 to 37.95)	0.93 (0.89 to 0.97)
High SDI	475,234 (449,623 to 489,570)	42.79 (40.55 to 44.07)	839,755 (764,451 to 885,156)	40.52 (37.45 to 42.45)	−0.25 (−0.35 to −0.16)
GBD region					
High-income Asia Pacific	79,543 (75,502 to 82,394)	39.72 (37.43 to 41.17)	207,277 (179,498 to 223,332)	44.89 (40.2 to 47.85)	0.33 (0.23 to 0.43)
Central Asia	6,180 (5,774 to 6,592)	12.9 (12.03 to 13.79)	8,893 (7,942 to 9,809)	10.82 (9.69 to 11.9)	−0.15 (−0.35 to 0.05)
East Asia	165,083 (142,142 to 189,658)	19.08 (16.55 to 21.79)	684,927 (559,523 to 823,301)	31.6 (25.9 to 37.85)	1.75 (1.66 to 1.84)
South Asia	28,138 (24,016 to 31,934)	4.69 (4 to 5.34)	85,115 (76,614 to 95,248)	5.65 (5.08 to 6.3)	0.46 (0.33 to 0.59)
Southeast Asia	29,321 (24,900 to 33,179)	11.28 (9.64 to 12.72)	116,942 (101,260 to 132,256)	17.7 (15.41 to 19.89)	1.45 (1.4 to 1.5)
Australasia	11,843 (10,910 to 12,804)	50.58 (46.64 to 54.65)	23,281 (20,502 to 26,413)	43.97 (38.91 to 49.55)	−0.58 (−0.71 to −0.45)
Caribbean	6,207 (5,825 to 6,607)	24.09 (22.57 to 25.67)	18,481 (16,074 to 20,936)	34.33 (29.87 to 38.86)	1.26 (1.19 to 1.34)
Central Europe	42,182 (40,230 to 43,856)	28.32 (27.03 to 29.45)	85,867 (79,290 to 92,787)	38.82 (35.71 to 41.96)	0.98 (0.82 to 1.14)
Eastern Europe	71,751 (69,017 to 73,943)	25.52 (24.5 to 26.32)	113,252 (104,415 to 122,489)	32.11 (29.59 to 34.73)	0.62 (0.51 to 0.73)
Western Europe	243,931 (229,598 to 253,378)	41.81 (39.51 to 43.4)	375,462 (337,713 to 401,850)	40.54 (37.17 to 43.07)	−0.11 (−0.26 to 0.04)
Andean Latin America	1902 (1,648 to 2,186)	9.49 (8.24 to 10.87)	8,452 (6,699 to 10,526)	14.39 (11.39 to 17.89)	1.43 (1.33 to 1.53)
Central Latin America	7,632 (7,311 to 7,896)	9.32 (8.9 to 9.67)	44,552 (39,665 to 49,721)	17.74 (15.75 to 19.81)	2.05 (1.99 to 2.11)
Southern Latin America	10,983 (10,130 to 11,892)	24.09 (22.12 to 26.13)	24,747 (21,922 to 27,561)	28.32 (25.1 to 31.53)	0.73 (0.56 to 0.9)
Tropical Latin America	9,843 (9,351 to 10,324)	11 (10.32 to 11.56)	44,245 (40,859 to 47,144)	17.17 (15.82 to 18.31)	1.42 (1.31 to 1.54)
North Africa and Middle East	17,568 (14,963 to 19,790)	10.4 (9.08 to 11.63)	66,087 (58,130 to 74,936)	14.43 (12.67 to 16.35)	1.31 (1.16 to 1.47)
High-income North America	167,645 (155,818 to 174,737)	47.34 (44.19 to 49.26)	244,681 (226,550 to 256,377)	38.75 (36.13 to 40.48)	−0.8 (−0.92 to −0.67)
Oceania	197 (162 to 239)	6.82 (5.75 to 8.03)	482 (410 to 560)	6.44 (5.55 to 7.42)	−0.18 (−0.27 to −0.1)
Central Sub-Saharan Africa	1,591 (1,268 to 1964)	7.4 (6.06 to 9)	4,206 (3,206 to 5,574)	7.87 (6.09 to 10.53)	0.24 (0.06 to 0.41)
Eastern Sub-Saharan Africa	8,179 (6,323 to 9,308)	11.08 (8.75 to 12.51)	17,952 (15,710 to 20,779)	11.23 (9.84 to 12.77)	−0.1 (−0.21 to 0.01)
Southern Sub-Saharan Africa	2,516 (2,234 to 3,057)	9.46 (8.37 to 11.65)	7,623 (6,875 to 8,472)	13.45 (12.16 to 14.83)	1.29 (1.07 to 1.51)
Western Sub-Saharan Africa	4,347 (3,709 to 5,101)	5.2 (4.44 to 6.04)	11,620 (9,673 to 13,673)	6.29 (5.36 to 7.3)	0.78 (0.71 to 0.85)

### 3.2 In metabolic risks associated with CRC, high BMI is the most critical risk factor

Metabolic risk factors accounted for 16.6% of the global CRC deaths burden in 2021, of which high BMI and high fasting glucose were the main contributors, accounting for 9.5 and 7.9%, respectively. This represents a significant increase compared to 1990, indicating that metabolic risk factors, specifically high BMI, have become important contributors to CRC risk (Supplementary Figure S1A). The CRC DALYs burden attributable to metabolic risk factors mirrored the same pattern of the burden of deaths (Supplementary Figure S1B). In 2021, the CRC burden of deaths and DALYs attributable to metabolic risk factors was higher in high SDI, at 18.9 and 19.1%, with the most prominent contribution from high BMI, at 10.9 and 11.6% (Supplementary Figures S1A,B). Within the GBD region, high-income North America had the highest burden, with high BMI also playing a significant role (Supplementary Figures S1A,B). These findings clearly indicate that high BMI is the most important risk factor among the metabolic risks associated with CRC.

### 3.3 Global burden of CRC deaths attributed to high BMI

**Figure 1:**
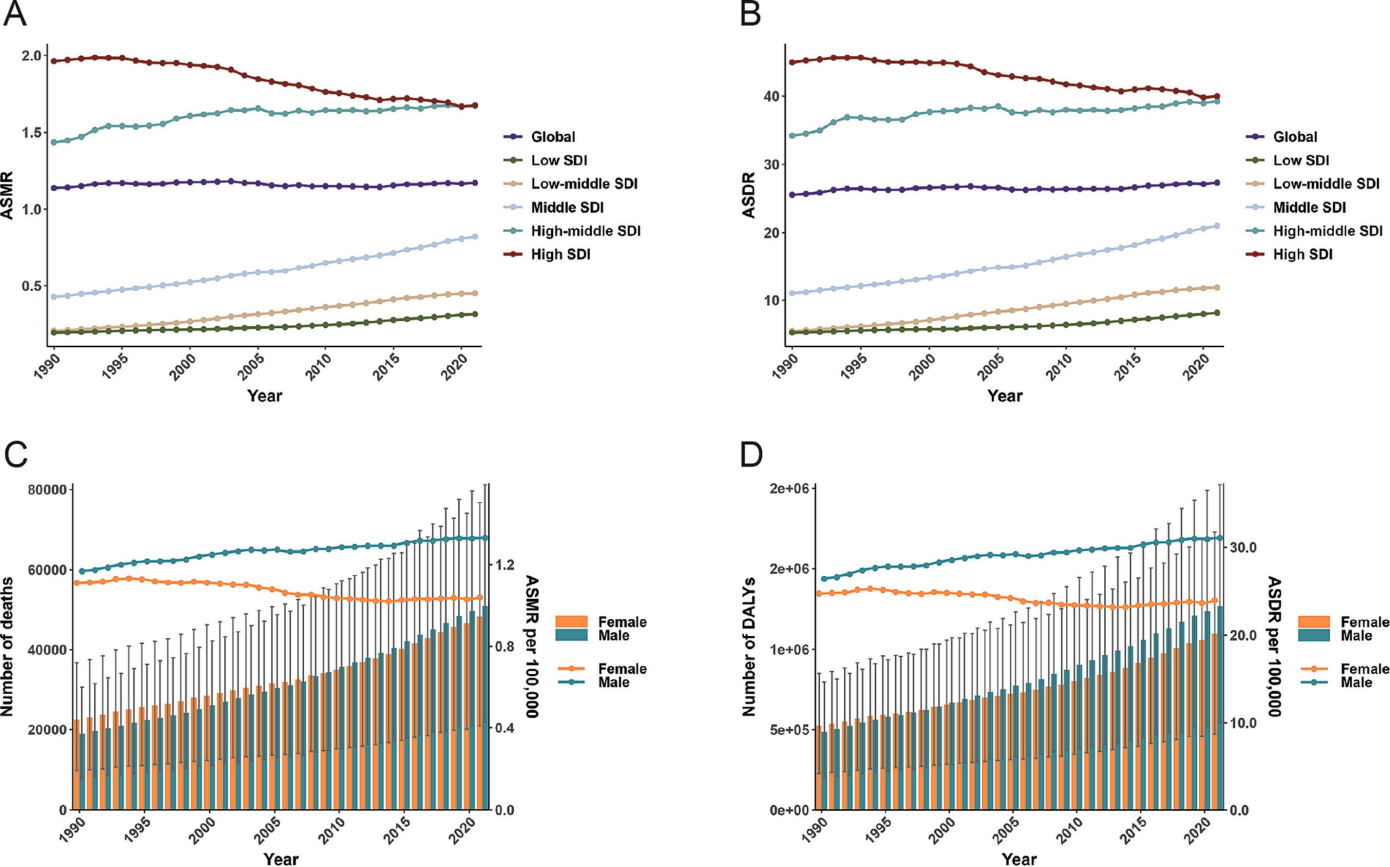
ASMR (A) and ASDR (B) for colorectal cancer attributable to high BMI by 5 SDI regions; the global number of deaths and ASMR for colorectal cancer attributable to high BMI by sex (C). The global number of DALYs and ASDR for colorectal cancer attributable to high BMI by sex (D); ASMR, age-standardized mortality rate; DALYs, disability-adjusted life-years; ASDR, age-standardized DALYs rate; SDI, sociodemographic index; BMI, body-mass index.

Over the past 32 years, the number of deaths due to CRC associated with high BMI has rapidly increased from 41,536 (95% UI: 17,666–67,379) in 1990 to 99,268 (95% UI: 42,956–157,949) in 2021, an increase of 139%. Meanwhile, ASMR has also increased, but the change is non-significant, rising from 1.14 per 100,000 population (95% UI: 0.48–1.86) in 1990 to 1.17 per 100,000 population (95% UI: 0.51–1.87) in 2021. The EAPC of ASMR was 0 (95% UI: −0.04 to 0.04), revealing a relatively stable trend (Table 2). From 1990 to 2021, the number of deaths from high BMI-related CRC increased significantly in men and women. The number of deaths among women was higher than that of men from 1990 to 2008; however, after 2008, the number of deaths among men surpassed that of women. Moreover, the ASMR in men has always been higher than that in women (Figure 1C). Although the number of deaths for men and women increased over the past 32 years, the ASMR for women revealed a significant downward trend (EAPC = −0.37, 95% CI: −0.42 to −0.31), while the ASMR for men depicted an upward trend (EAPC = 0.39, 95% CI: 0.36–0.42) (Table 2; Figure 1C).

**Table 2:** Deaths of colorectal cancer attributable to high BMI in 1990 and 2021 by sex, SDI, and GBD region, with estimated annual percentage change from 1990 to 2021.

Deaths of colorectal cancer attributable to high BMI in 1990 and 2021 by sex, SDI, and GBD region, with estimated annual percentage change from 1990 to 2021					
Characteristics	Deaths cases in 1990	ASMR per100,000 in 1990	Deaths cases in 2021	ASMR per100,000 in 2021	EAPC (1990–2021)
Global	41,536 (17,666 to 67,379)	1.14 (0.48 to 1.86)	99,268 (42,956 to 157,949)	1.17 (0.51 to 1.87)	0 (−0.04 to 0.04)
Sex					
Male	18,949 (7,915 to 30,723)	1.17 (0.49 to 1.9)	50,976 (21,914 to 81,236)	1.33 (0.57 to 2.13)	0.39 (0.36 to 0.42)
Female	22,586 (9,703 to 36,765)	1.11 (0.48 to 1.81)	48,292 (20,914 to 76,782)	1.04 (0.45 to 1.65)	−0.37 (−0.42 to −0.31)
SDI					
Low SDI	442 (161 to 737)	0.2 (0.07 to 0.33)	1,567 (615 to 2,540)	0.32 (0.12 to 0.52)	1.5 (1.38 to 1.62)
Low-middle SDI	1,244 (472 to 1982)	0.21 (0.08 to 0.33)	6,391 (2,690 to 10,103)	0.45 (0.19 to 0.71)	2.72 (2.65 to 2.79)
Middle SDI	4,237 (1,566 to 6,975)	0.43 (0.16 to 0.7)	21,654 (9,250 to 34,503)	0.82 (0.35 to 1.32)	2.13 (2.11 to 2.16)
High-middle SDI	13,680 (5,865 to 22,168)	1.44 (0.61 to 2.33)	32,966 (14,288 to 52,368)	1.67 (0.72 to 2.66)	0.4 (0.32 to 0.48)
High SDI	21,852 (9,256 to 35,533)	1.96 (0.83 to 3.19)	36,530 (15,670 to 58,139)	1.68 (0.73 to 2.66)	−0.64 (−0.69 to −0.59)
GBD region					
High-income Asia Pacific	1,425 (521 to 2,304)	0.73 (0.27 to 1.17)	4,240 (1,633 to 6,744)	0.83 (0.32 to 1.31)	0.32 (0.27 to 0.36)
Central Asia	488 (202 to 784)	1.04 (0.43 to 1.68)	769 (329 to 1,215)	0.96 (0.41 to 1.53)	0.1 (−0.03 to 0.22)
East Asia	3,773 (1,309 to 6,406)	0.45 (0.16 to 0.77)	20,371 (8,475 to 33,799)	0.96 (0.4 to 1.59)	2.41 (2.34 to 2.48)
South Asia	517 (173 to 827)	0.09 (0.03 to 0.14)	3,043 (1,182 to 4,777)	0.2 (0.08 to 0.32)	2.81 (2.76 to 2.86)
Southeast Asia	653 (221 to 1,050)	0.25 (0.08 to 0.4)	4,057 (1,623 to 6,569)	0.62 (0.25 to 1)	3.04 (2.93 to 3.15)
Australasia	561 (233 to 894)	2.41 (1 to 3.83)	1,123 (476 to 1797)	2.01 (0.85 to 3.2)	−0.78 (−0.86 to −0.71)
Caribbean	244 (102 to 387)	0.97 (0.4 to 1.54)	841 (355 to 1,385)	1.55 (0.66 to 2.56)	1.68 (1.62 to 1.73)
Central Europe	3,628 (1,590 to 5,836)	2.46 (1.08 to 3.96)	6,941 (3,093 to 11,133)	3.03 (1.35 to 4.85)	0.56 (0.42 to 0.69)
Eastern Europe	5,381 (2,332 to 8,579)	1.92 (0.83 to 3.06)	9,052 (3,878 to 14,440)	2.53 (1.09 to 4.04)	0.72 (0.6 to 0.84)
Western Europe	12,481 (5,253 to 20,402)	2.11 (0.89 to 3.45)	18,026 (7,623 to 29,686)	1.77 (0.75 to 2.9)	−0.65 (−0.71 to −0.59)
Andean Latin America	134 (55 to 221)	0.67 (0.28 to 1.12)	672 (287 to 1,131)	1.15 (0.49 to 1.93)	1.81 (1.69 to 1.94)
Central Latin America	530 (223 to 853)	0.67 (0.28 to 1.07)	3,149 (1,403 to 5,105)	1.26 (0.56 to 2.06)	2.11 (2.04 to 2.19)
Southern Latin America	933 (403 to 1,521)	2.07 (0.89 to 3.36)	2,235 (992 to 3,642)	2.52 (1.12 to 4.11)	0.9 (0.73 to 1.08)
Tropical Latin America	699 (290 to 1,136)	0.8 (0.33 to 1.29)	3,630 (1,534 to 5,850)	1.42 (0.6 to 2.29)	1.87 (1.76 to 1.98)
North Africa and Middle East	1,341 (566 to 2,184)	0.83 (0.35 to 1.35)	5,637 (2,451 to 8,982)	1.31 (0.58 to 2.1)	1.7 (1.54 to 1.85)
High-income North America	8,084 (3,450 to 13,011)	2.27 (0.97 to 3.65)	12,769 (5,673 to 19,870)	1.95 (0.87 to 3.03)	−0.66 (−0.76 to −0.55)
Oceania	15 (6 to 25)	0.51 (0.21 to 0.85)	43 (18 to 70)	0.59 (0.25 to 0.96)	0.51 (0.42 to 0.6)
Central Sub-Saharan Africa	50 (19 to 86)	0.23 (0.09 to 0.39)	254 (96 to 438)	0.49 (0.18 to 0.83)	2.41 (2.24 to 2.57)
Eastern Sub-Saharan Africa	209 (73 to 350)	0.28 (0.1 to 0.46)	774 (295 to 1,284)	0.48 (0.18 to 0.8)	1.62 (1.52 to 1.72)
Southern Sub-Saharan Africa	197 (83 to 310)	0.77 (0.33 to 1.22)	808 (341 to 1,270)	1.49 (0.63 to 2.34)	2.32 (2.06 to 2.58)
Western Sub-Saharan Africa	194 (76 to 313)	0.24 (0.09 to 0.38)	834 (336 to 1,365)	0.47 (0.19 to 0.75)	2.39 (2.33 to 2.45)

Significant differences in the burden of CRC were caused by high BMI in regions with different SDI levels. In 2021, the high SDI regions had the highest number of deaths from high BMI-related CRC, with 36,530 (95% UI: 15,670–58,139), accounting for more than 35% of global cases (Table 2). Although the ASMR in high SDI regions decreased from 1.96 per 100,000 population (95% UI: 0.83–3.19) in 1990 to 1.68 per 100,000 population (95% UI: 0.73–2.66) in 2021, it was still significantly higher than that in other SDI regions. Over the past 32 years, the ASMR in low, low-middle, middle, and high-middle SDI regions revealed an upward trend, with the most significant increase in low-middle SDI regions (EAPC = 2.72, 95% CI: 2.65–2.79). Conversely, the ASMR in the high SDI regions significantly decreased (EAPC = −0.64, 95% CI: −0.69 to −0.59) (Table 2; Figure 1A).

At the GBD regional level, in 2021, East Asia had the highest number of deaths from high BMI-related CRC, with 20,371 (95% UI: 8,475–33,799), followed by Western Europe, with 18,026 (95% UI: 7,623–29,686). Central Europe had the highest ASMR, at 3.03 per 100,000 population (95% UI: 1.35–4.85). From 1990 to 2021, Southeast Asia had the most significant increase in ASMR (EAPC = 3.04, 95% CI: 2.93–3.15). Furthermore, Australasia experienced the largest decrease in ASMR (EAPC = −0.78, 95% CI: −0.86 to −0.71) (Table 2).

**Figure 2:**
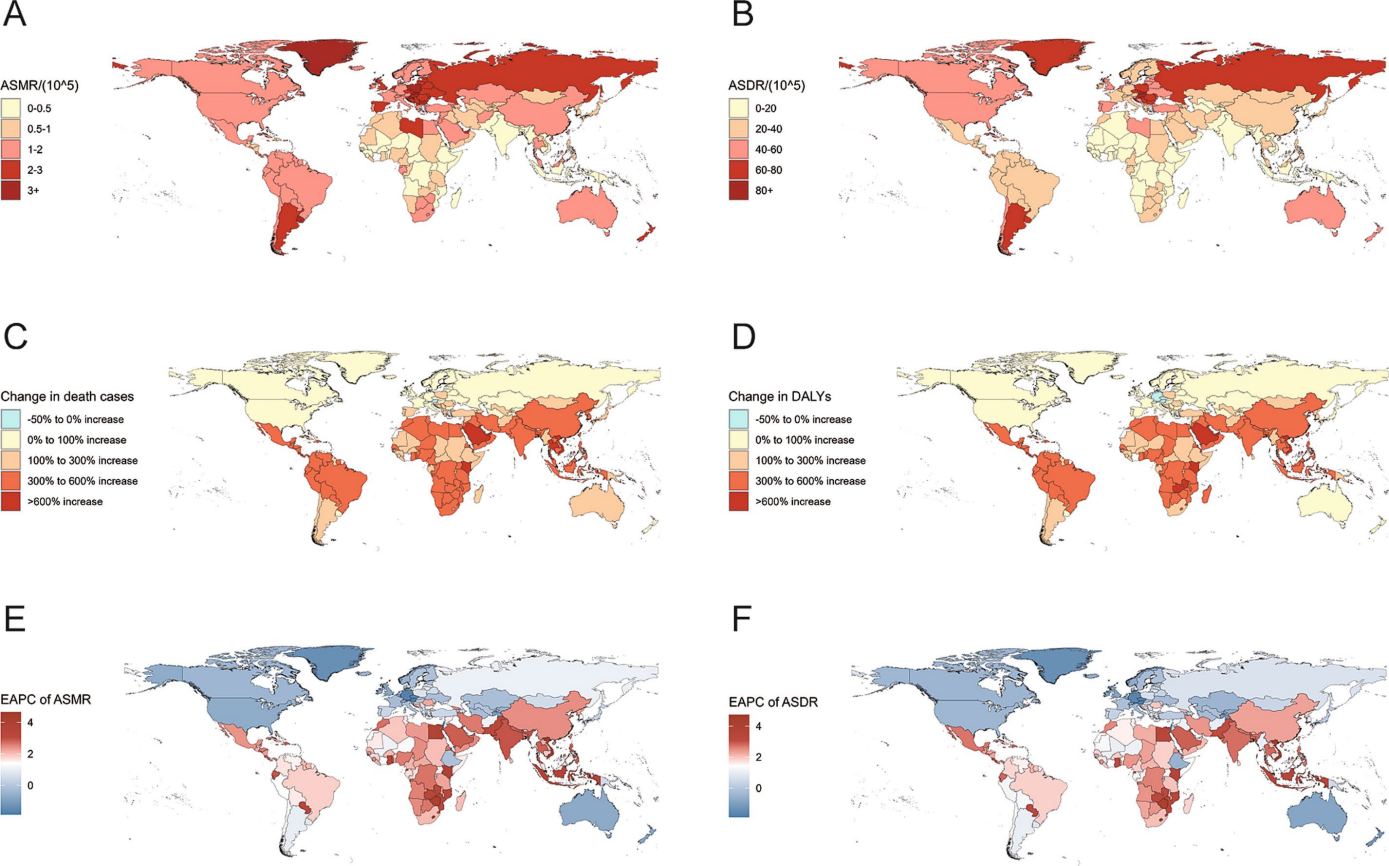
ASMR (A) and ASDR (B) for colorectal cancer attributable to high BMI in 204 countries in 2021; the percentage change in the number of deaths (C) and DALYs (D) for colorectal cancer attributable to high BMI from 1990 to 2021. The EAPC of the ASMR (E) and ASDR (F) for colorectal cancer attributable to high BMI from 1990 to 2021; ASMR, age-standardized mortality rate; ASDR, age-standardized DALYs rate; DALYs, disability-adjusted life-years; BMI, body-mass index; EAPC, estimated annual percentage change.

At the national level, in 2021, China had the highest number of deaths from CRC due to high BMI, with 19,418 (95% UI: 8,053–32,452), followed by the United States, with 11,402 (95% UI: 5,070–17,661). Kuwait had the largest increase in the number of deaths, with a 974.3% rise over 32 years. Among the 204 countries, only Austria saw a decrease in the number of deaths (Supplementary Table S1; Figure 2C). Hungary had the highest ASMR, at 3.79 per 100,000 population (95% UI: 1.72–6.19), followed by Slovakia and Uruguay, with ASMRs of 3.53 per 100,000 population (95% UI: 1.61–5.66) and 3.45 per 100,000 population (95% UI: 1.54–5.72), respectively (Supplementary Table S1; Figure 2A). Moreover, since 1990, Vietnam has experienced the most significant increase in ASMR (EAPC = 4.65, 95% CI: 4.49–4.81). Meanwhile, 30 countries revealed a downward trend in ASMR, with Austria experiencing the most significant decrease (EAPC = −1.86, 95% CI: −1.92 to −1.8) (Supplementary Table S1; Figure 2E).

### 3.4 Global burden of CRC DALYs attributed to high BMI

From 1990 to 2021, the number of DALYs due to high BMI-related CRC increased from 1,015,042 (95% UI: 429,787–1,631,974) in 1990 to 2,364,664 (95% UI: 1,021,594–3,752,340) in 2021 globally, an increase of 133%. The ASDR also rose from 25.54 per 100,000 population (95% UI: 10.83–41.2) in 1990 to 27.33 per 100,000 population (95% UI: 11.8–43.37) in 2021, with an EAPC of 0.12 (95% CI: 0.08–0.16) (Table 3). The trend of DALYs due to high BMI-related CRC in both men and women was similar to the trend in mortality. In 1990, the number of DALYs in women was higher than in men, but by 1999, the number of DALYs in men surpassed that in women, and the gap continued to widen (Figure 1D). Additionally, the ASDR for men increased from 26.42 per 100,000 population (95% UI: 10.97–42.88) in 1990 to 31.09 per 100,000 population (95% UI: 13.36–49.47) in 2021, revealing a significant upward trend (EAPC = 0.48, 95% CI: 0.45–0.51). In contrast, the ASDR for women decreased from 24.73 per 100,000 population (95% UI: 10.59–39.99) in 1990 to 23.96 per 100,000 population (95% UI: 10.36–37.75) in 2021, exhibiting a significant downward trend (EAPC = −0.26, 95% CI: −0.31 to −0.2) (Table 3; Figure 1D).

**Table 3:** DALYs of colorectal cancer attributable to high BMI in 1990 and 2021 by sex, SDI, and GBD region, with estimated annual percentage change from 1990 to 2021.

DALYs of colorectal cancer attributable to high BMI in 1990 and 2021 by sex, SDI, and GBD region, with estimated annual percentage change from 1990 to 2021					
Characteristics	DALYs in 1990	ASDR per 100,000 in 1990	DALYs in 2021	ASDR per 100,000 in 2021	EAPC (1990–2021)
Global	1,015,042 (429,787 to 1,631,974)	25.54 (10.83 to 41.2)	2,364,664 (1,021,594 to 3,752,340)	27.33 (11.8 to 43.37)	0.12 (0.08 to 0.16)
Sex					
Male	489,266 (202,595 to 796,351)	26.42 (10.97 to 42.88)	1,268,891 (544,822 to 2,022,285)	31.09 (13.36 to 49.47)	0.48 (0.45 to 0.51)
Female	525,776 (225,046 to 849,420)	24.73 (10.59 to 39.99)	1,095,773 (474,290 to 1,727,956)	23.96 (10.36 to 37.75)	−0.26 (−0.31 to −0.2)
SDI					
Low SDI	13,400 (4,984 to 22,375)	5.28 (1.94 to 8.78)	47,047 (18,740 to 75,740)	8.17 (3.23 to 13.2)	1.33 (1.22 to 1.45)
Low-middle SDI	37,581 (14,269 to 60,129)	5.52 (2.1 to 8.81)	184,448 (77,502 to 290,094)	11.87 (5 to 18.73)	2.69 (2.63 to 2.76)
Middle SDI	125,728 (46,880 to 206,617)	11.03 (4.1 to 18.13)	584,511 (248,469 to 929,608)	21.01 (8.93 to 33.45)	2.1 (2.07 to 2.14)
High-middle SDI	346,229 (147,489 to 559,453)	34.2 (14.56 to 55.28)	769,290 (332,395 to 1,220,598)	39.23 (16.94 to 62.34)	0.31 (0.24 to 0.37)
High SDI	490,183 (210,225 to 788,177)	44.94 (19.27 to 72.19)	775,809 (337,834 to 1,225,934)	40 (17.48 to 62.93)	−0.48 (−0.52 to −0.43)
GBD region					
High-income Asia Pacific	36,686 (13,456 to 59,451)	17.92 (6.55 to 28.98)	80,601 (31,642 to 127,988)	19.32 (7.65 to 30.53)	0.13 (0.09 to 0.18)
Central Asia	14,012 (5,760 to 22,453)	28.36 (11.66 to 45.42)	21,393 (9,093 to 33,880)	24.46 (10.4 to 38.7)	−0.22 (−0.32 to −0.13)
East Asia	113,878 (39,419 to 193,736)	11.93 (4.13 to 20.29)	529,352 (219,133 to 886,681)	24.42 (10.09 to 40.83)	2.31 (2.21 to 2.41)
South Asia	16,694 (5,703 to 26,476)	2.47 (0.83 to 3.93)	91,500 (35,883 to 144,388)	5.67 (2.22 to 8.92)	2.74 (2.69 to 2.78)
Southeast Asia	20,953 (7,311 to 33,668)	7.05 (2.42 to 11.33)	119,340 (48,437 to 193,339)	16.64 (6.73 to 26.92)	2.84 (2.72 to 2.97)
Australasia	13,424 (5,580 to 21,236)	58.17 (24.18 to 91.85)	23,565 (10,170 to 37,480)	46.65 (20.18 to 74.39)	−0.94 (−1.02 to −0.85)
Caribbean	6,308 (2,656 to 10,112)	23.81 (10 to 38.23)	20,386 (8,687 to 33,783)	37.94 (16.17 to 62.94)	1.66 (1.61 to 1.71)
Central Europe	87,450 (38,342 to 140,811)	57.78 (25.32 to 93)	148,738 (66,397 to 238,496)	68.94 (30.82 to 110.6)	0.48 (0.34 to 0.61)
Eastern Europe	137,909 (59,625 to 219,011)	48.61 (20.98 to 77.14)	208,724 (90,210 to 331,586)	60 (25.94 to 95.33)	0.45 (0.31 to 0.58)
Western Europe	264,280 (111,569 to 431,654)	46.8 (19.73 to 76.51)	344,976 (147,807 to 564,628)	39.08 (16.77 to 63.51)	−0.67 (−0.74 to −0.6)
Andean Latin America	3,674 (1,506 to 5,995)	16.83 (6.89 to 27.44)	16,947 (7,444 to 28,548)	28 (12.28 to 47.21)	1.67 (1.55 to 1.79)
Central Latin America	14,473 (6,124 to 23,291)	16.13 (6.82 to 25.96)	84,537 (37,822 to 134,896)	32.79 (14.66 to 52.33)	2.32 (2.25 to 2.4)
Southern Latin America	22,072 (9,493 to 36,141)	47.48 (20.42 to 77.6)	50,170 (22,346 to 81,468)	58.65 (26.14 to 95.15)	0.95 (0.8 to 1.11)
Tropical Latin America	19,341 (8,092 to 31,334)	19.74 (8.2 to 31.96)	94,304 (39,800 to 150,658)	36.04 (15.2 to 57.63)	1.89 (1.79 to 2)
North Africa and Middle East	39,568 (16,500 to 65,046)	21.25 (8.93 to 34.73)	156,482 (66,565 to 247,802)	31.83 (13.68 to 50.63)	1.46 (1.32 to 1.6)
High-income North America	185,197 (80,214 to 295,440)	54.48 (23.66 to 86.76)	296,820 (135,095 to 460,709)	49.52 (22.63 to 76.52)	−0.42 (−0.5 to −0.33)
Oceania	471 (194 to 788)	13.6 (5.52 to 22.78)	1,362 (562 to 2,191)	15.48 (6.43 to 25.08)	0.44 (0.37 to 0.52)
Central Sub-Saharan Africa	1,494 (555 to 2,560)	5.96 (2.23 to 10.14)	7,638 (2,875 to 13,168)	12.19 (4.62 to 21.03)	2.34 (2.19 to 2.5)
Eastern Sub-Saharan Africa	6,359 (2,271 to 10,622)	7.52 (2.67 to 12.61)	23,026 (8,703 to 37,649)	11.97 (4.57 to 19.8)	1.33 (1.23 to 1.43)
Southern Sub-Saharan Africa	5,488 (2,316 to 8,602)	18.78 (7.96 to 29.48)	21,915 (9,192 to 34,323)	35.73 (15.05 to 55.83)	2.36 (2.1 to 2.62)
Western Sub-Saharan Africa	5,311 (2,113 to 8,559)	5.71 (2.26 to 9.23)	22,886 (9,128 to 37,859)	10.79 (4.35 to 17.69)	2.2 (2.14 to 2.25)

In terms of SDI, from 1990 to 2021, the number of DALYs and ASDR due to high BMI-related CRC in high SDI regions has always been the highest. However, the ASDR in high SDI regions decreased from 44.94 per 100,000 population (95% UI: 19.27–72.19) in 1990 to 40 per 100,000 population (95% UI: 17.48–62.93) in 2021, with an EAPC of −0.48 (95% CI: −0.52 to −0.43). This is the only SDI region where the ASDR exhibited a downward trend. The ASDR in other SDI regions revealed an upward trend, with the most significant increase in low-middle SDI regions (EAPC = 2.69, 95% CI: 2.63–2.76) (Table 3; Figure 1B).

For the GBD regions, in 2021, East Asia recorded the largest number of DALYs owing to high BMI-related CRC, with 529,352 cases (95% UI: 219,133–886,681). Central Europe had the most significant ASDR at 68.94 per 100,000 population (95% UI: 30.82–110.6). Over the past 32 years, 17 out of 21 GBD regions revealed an upward trend in ASDR, with Southeast Asia experiencing the most notable increase (EAPC = 2.84, 95% UI: 2.72–2.97). Conversely, Western Europe, high-income North America, Central Asia, and Australasia exhibited a downward trend in ASDR, with Australasia having the most pronounced decrease (EAPC = −0.94, 95% CI: −1.02 to −0.85) (Table 3).

From a national perspective, in 2021, China had the highest number of DALYs due to high BMI-related CRC, with 507,316 (95% UI: 209,264–853,770). From 1990 to 2021, among 204 countries, the number of DALYs increased rapidly in most countries, while only Austria, Germany, and the Czech Republic observed a decrease in DALYs, with reductions of 13.9, 5.6 and 4.8%, respectively (Supplementary Table S2; Figure 2D). Hungary exhibited the highest ASDR, at 92.03 per 100,000 population (95% UI: 41.88–149.98), followed by Slovakia, at 82.15 per 100,000 population (95% UI: 37.4–131.78) (Supplementary Table S2; Figure 2B). Notably, more than half of the countries have seen an increase in the ASDR EAPC. Vietnam (EAPC = 4.74, 95% CI: 4.54–4.94), Lesotho (EAPC = 4.7, 95% CI: 4.15–5.25), and Zimbabwe (EAPC = 4.11, 95% CI: 3.45–4.78) had the largest increases, which may indicate severe health challenges in these countries. Conversely, the ASDR in a few countries, such as the Czech Republic (EAPC = −1.81, 95% CI: −1.99 to −1.63) and Germany (EAPC = −1.65, 95% CI: −1.77 to −1.54), exhibited the most significant decrease (Supplementary Table S2; Figure 2F).

### 3.5 Global CRC burden attributable to high BMI by age, sex, and SDI

**Figure 3:**
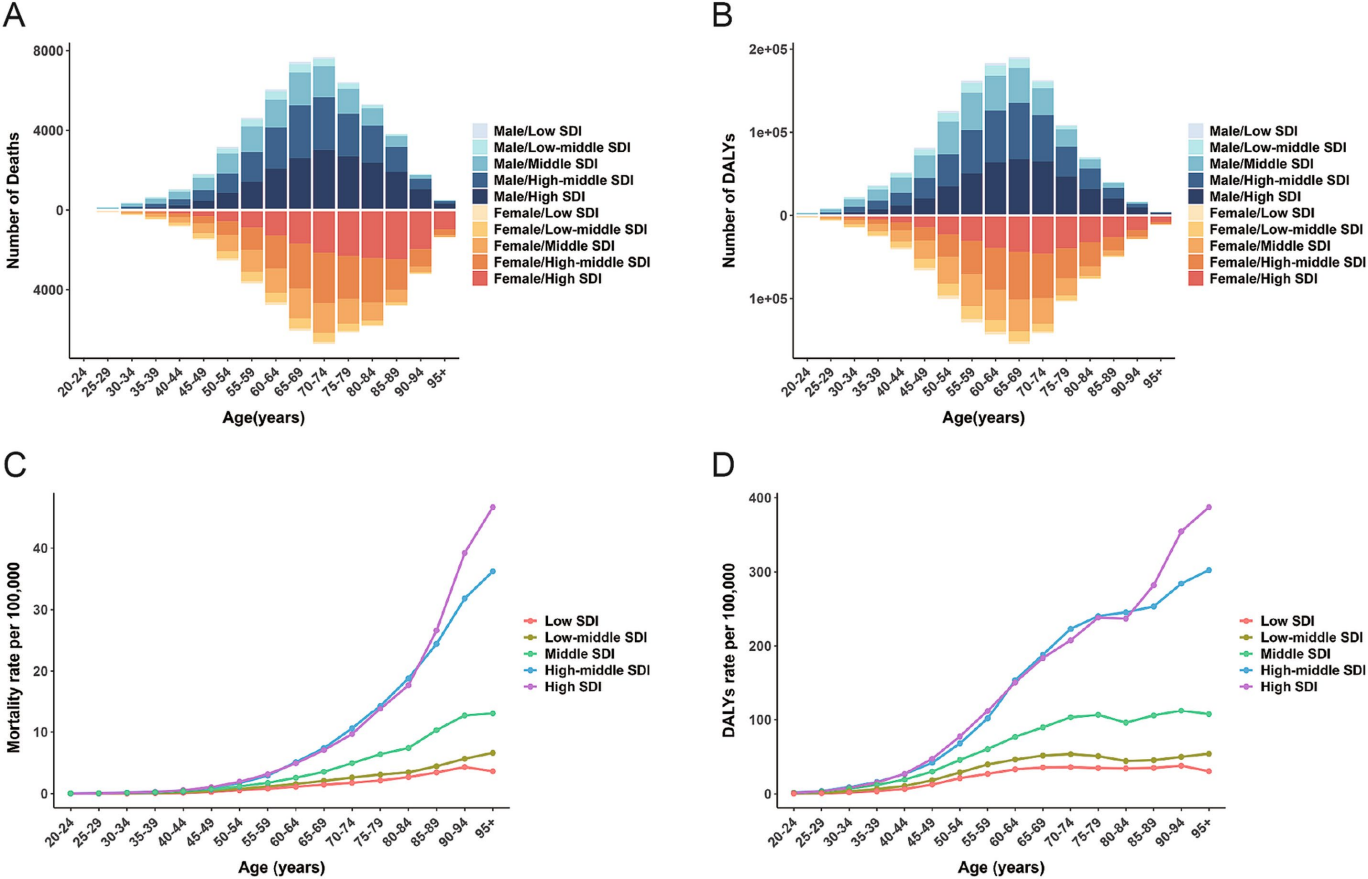
The global number of deaths (A) and DALYs (B) for colorectal cancer attributable to high BMI across age groups by sex and 5 SDI regions in 2021; The mortality rate (C) and DALYs rate (D) for colorectal cancer attributable to high BMI across age groups by 5 SDI regions in 2021; DALYs, disability-adjusted life-years; SDI, sociodemographic index; BMI, body-mass index.

**Figure 4:**
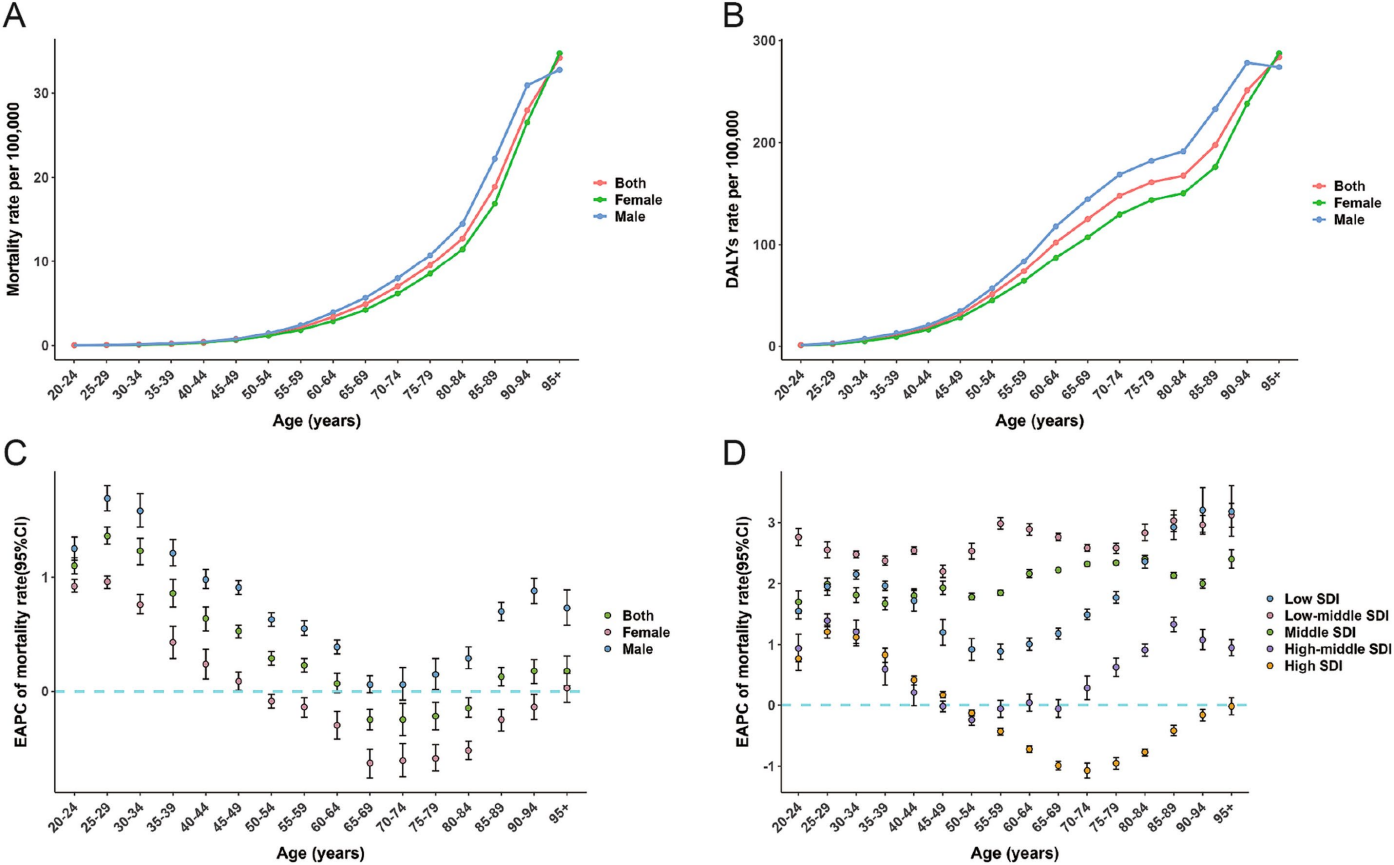
The mortality rate (A) and DALYs rate (B) for colorectal cancer attributable to high BMI across age groups by sex in 2021; EAPC of the mortality rate for colorectal cancer attributable to high BMI across age groups by sex from 1990-2021 (C); EAPC of the mortality rate for colorectal cancer attributable to high BMI across age groups by 5 SDI from 1990-2021 (D); DALYs, disability-adjusted life-years; SDI, sociodemographic index; BMI, body-mass index; EAPC, estimated annual percentage change.

In 2021, the total number of deaths due to high BMI-related CRC increased for both men and women, with male deaths reaching 50,976 and female deaths reaching 48,292 (Table 2). The number of deaths for men and women exhibited a synchronous pattern, first increasing and then decreasing with age, peaking at 70–74 years. However, male deaths were not always higher than female deaths. Before the age of 80–84 years, male deaths were significantly higher than female deaths, but this trend reversed after the age of 80–84 years (Figure 3A). The trend in mortality rates was similar, with age-specific mortality rates in men and women increasing with age. Female mortality rates significantly exceeded male rates at ages 95+, whereas male mortality rates were higher than female rates before this age range (Figure 4A). The age-specific trend for DALYs was similar to that for mortality, but the peak number of DALYs occurred in the 65–69 age group (Table 3; Figure 3B). The age-specific DALY rates for men and women increased significantly with age, but the DALY rate for men began to decline after 90–94 years of age (Figure 4B). Globally, from 1990 to 2021, age-specific mortality rates for men and women in the 20–49 and 95+ age groups increased, with the 25–29 age group experiencing the fastest growth in mortality rates. Among men, from 1990 to 2021, mortality rates increased across almost all age groups, with the 25–29 age group exhibiting the most significant increase. In contrast, among women, mortality rates decreased in the 50–94 years age group, with the 65–69 years age group revealing the most significant decrease (Figure 4C).

In 2021, across the five SDI regions, age-specific mortality rates due to high BMI-related CRC generally increased with age. Age-specific mortality rates in high and middle-high SDI regions were significantly higher than in other regions (Figure 3C)The age-specific DALYs rate was similar to the mortality rate, with the difference that the DALYs rates in the high, middle, low-middle, and low SDI regions depicted a declining trend at ages 80–84 (Figure 3D). The peak age-specific mortality and DALY rates in most regions occurred after the age of 90. Similarly, from 1990 to 2021, the age-specific mortality rates in the low, low-middle, and middle SDI regions increased significantly across all age groups. Among the five SDI regions, the low-middle SDI regions had the largest increase in mortality rates, followed by the middle SDI regions (Figure 4D). In high SDI regions, age-specific mortality rates in the 50–94 age group decreased annually, with the 70–74 age group experiencing the largest decline. In high-middle SDI regions, only the 50–54 age group’s age-specific mortality rate decreased, while mortality rates in relatively younger and older age groups increased (Figure 4D).

### 3.6 Association between SDI and the burden of CRC burden attributable to high BMI

**Figure 5:**
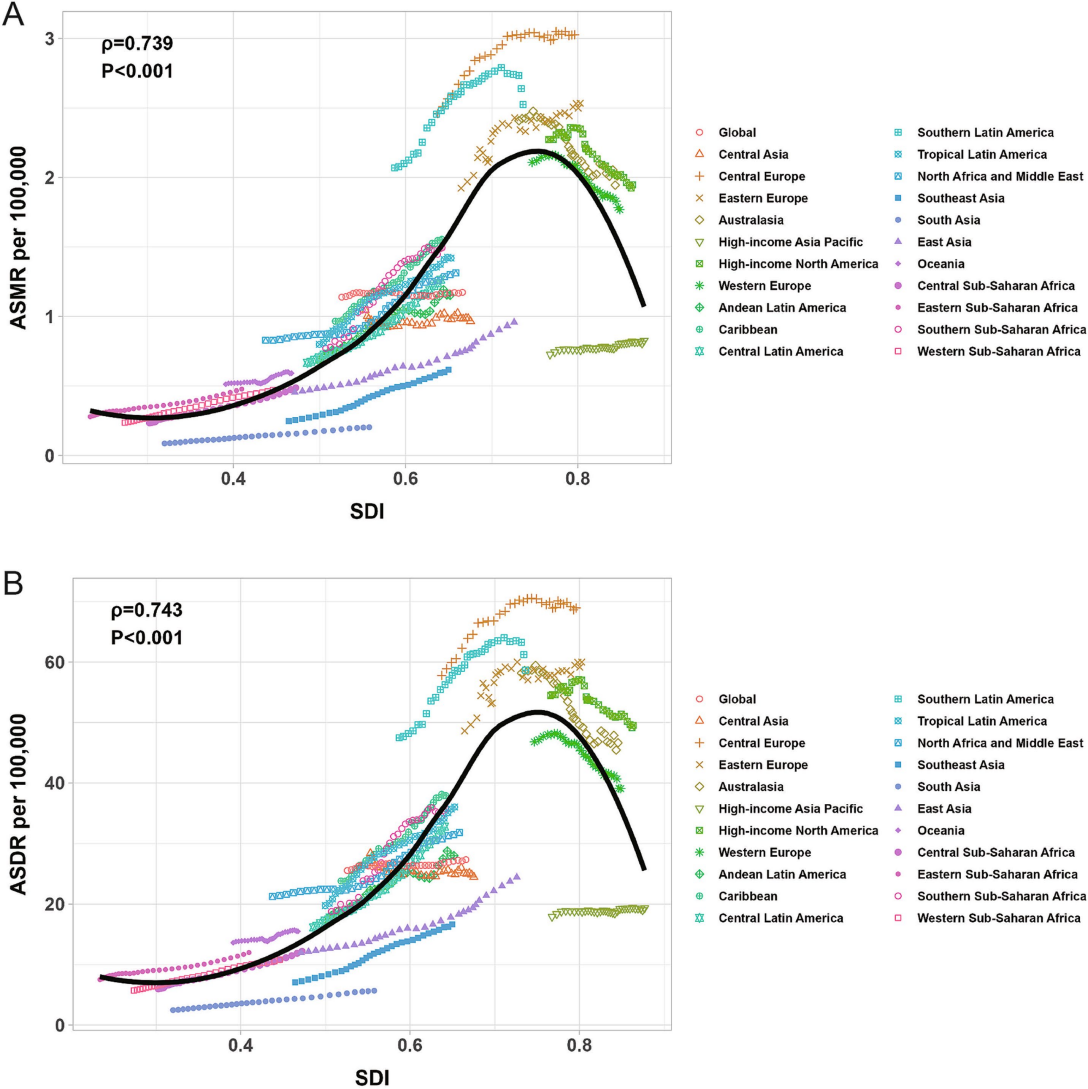
The correlation between ASMR for colorectal cancer attributable to high BMI and SDI (A); The correlation between ASDR for colorectal cancer attributable to high BMI and SDI (B); ASMR, age-standardized mortality rate; ASDR, age-standardized DALYs rate; SDI, sociodemographic index; BMI, body-mass index.

**Figure 6:**
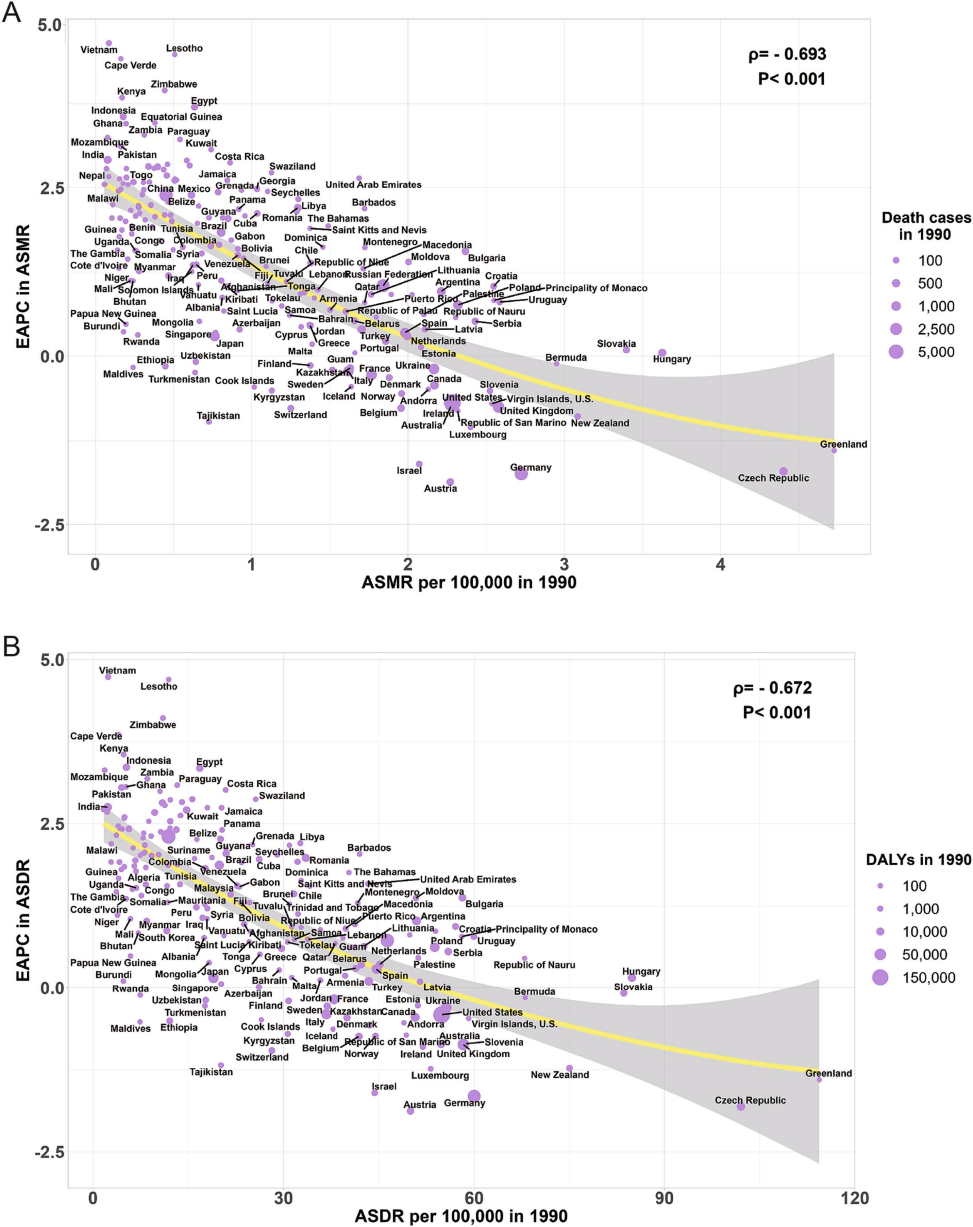
The correlation between ASMR for colorectal cancer attributable to high BMI in 1990 and EAPC of ASMR (A); The correlation between ASDR for colorectal cancer attributable to high BMI in 1990 and EAPC of ASDR (B); ASMR, age-standardized mortality rate; ASDR, age-standardized DALYs rate; BMI, body-mass index; EAPC, estimated annual percentage change.

**Figure 7:**
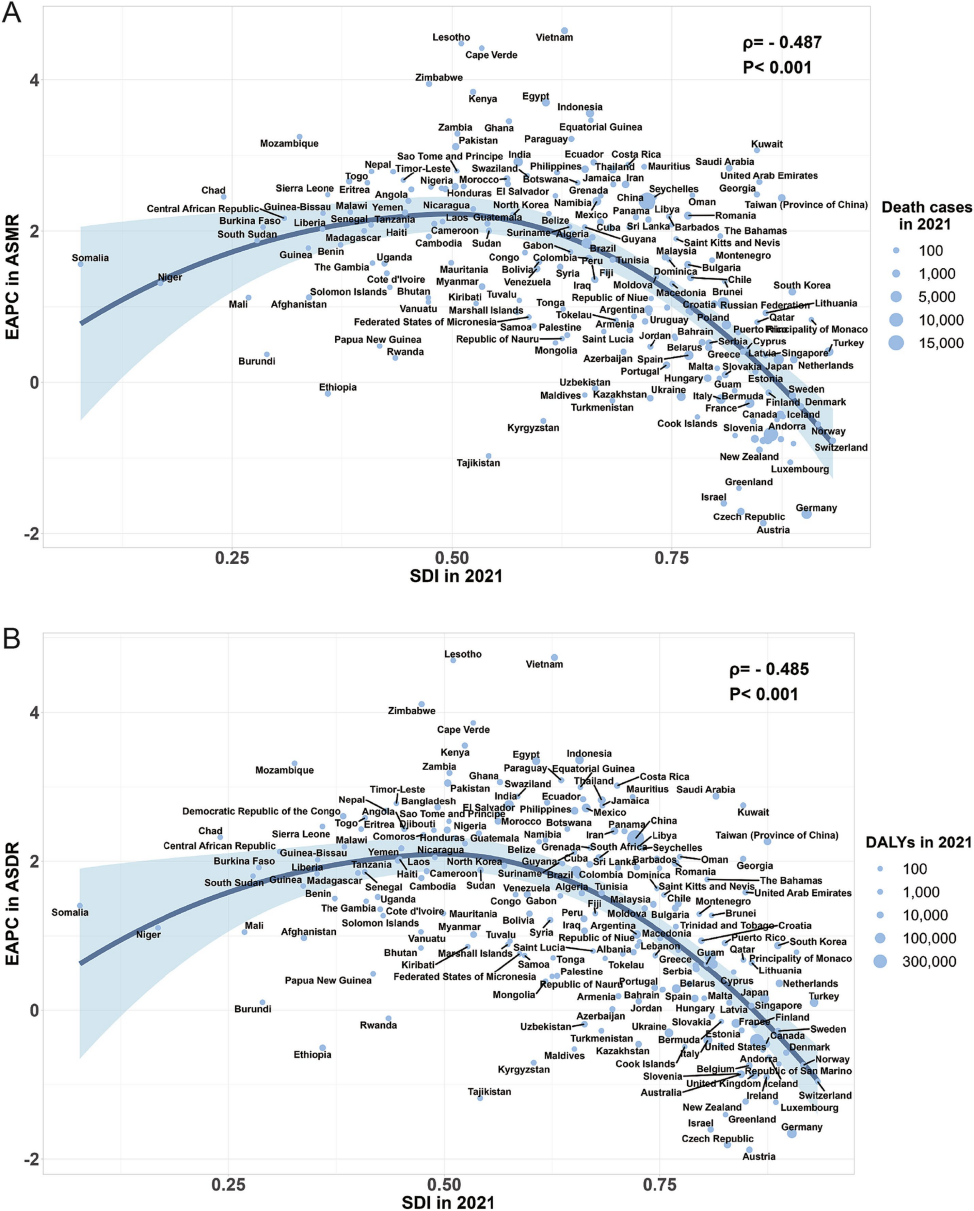
The correlation between the EAPC of ASMR for colorectal cancer attributable to high BMI and SDI in 2021 (A); The correlation between the EAPC of ASDR for colorectal cancer attributable to high BMI and SDI in 2021 (B); ASMR, age-standardized mortality rate; ASDR, age-standardized DALYs rate; SDI, sociodemographic index; BMI, body-mass index; EAPC, estimated annual percentage change.

Across the 21 GBD regions globally, from 1990 to 2021, the ASMR of high BMI-related CRC exhibited an inverted V-shaped nonlinear relationship with the SDI (*ρ* = 0.739, *p* < 0.001), peaking at an SDI of approximately 0.75. When SDI < 0.75, the ASMR increased gradually, whereas when SDI > 0.75, the ASMR declined rapidly (Figure 5A). A comparable relationship was also observed between the ASDR and SDI (Figure 5B). Among 204 countries worldwide, the ASMR of high BMI-related CRC in 1990 was significantly negatively correlated with EAPC from 1990 to 2021 (ρ = −0.693, *p* < 0.001) (Figure 6A), The ASDR in 1990 exhibited the same trend as the EAPC from 1990 to 2021 (ρ = −0.672, *p* < 0.001) (Figure 6B). The relationship between the EAPC of high BMI-related CRC and the SDI in 2021 exhibited an inverted “U” shape. When the SDI was below 0.5, a positive correlation was observed. When the SDI ≥ 0.5, both the EAPC of ASMR (ρ = −0.487, *p* < 0.001) and the EAPC of ASDR (ρ = −0.485, *p* < 0.001) were negatively correlated with the SDI (Figures 7A,B). The SDI for 204 countries from 1990 to 2021 are listed in Supplementary Table S3.

### 3.7 Prediction of age-standardized rate by autoregressive integrated moving average models

**Figure 8:**
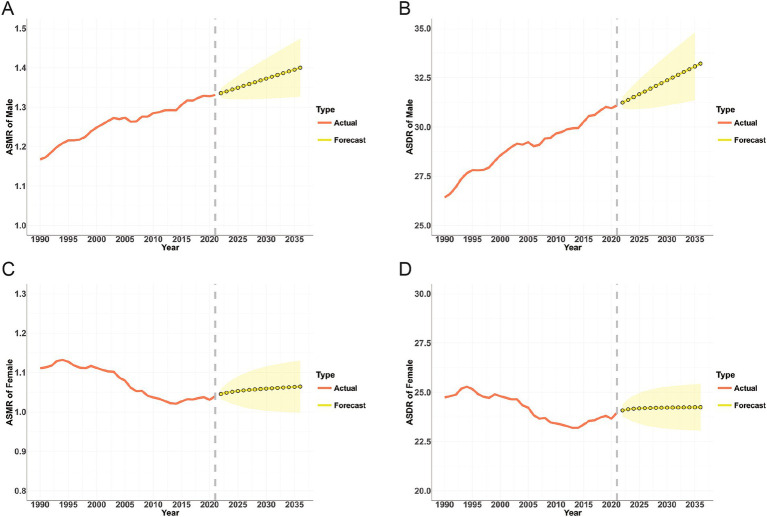
Projections of ASMR (A,C) and ASDR (B,D) for colorectal cancer attributable to high BMI in males and females from 2022 to 2035; The red line represents the actual value, and the fan represents the predicted distribution between the 2.5 and 97.5% quantiles. The forecast average is shown as a yellow dot. The vertical dotted line indicates where the prediction begins; ASMR, age-standardized mortality rate; ASDR, age-standardized DALYs rate; BMI, body-mass index.

According to the ARIMA model, from 2022 to 2035, the burden of high BMI-related CRC in males will continue to rise, with the ASMR increasing from 1.33 per 100,000 population (95% UI: 0.57–2.13) in 2021 to 1.4 per 100,000 population (95% UI: 1.33–1.47) in 2035, and the ASDR increasing from 31.09 per 100,000 population (95% UI: 13.36–49.47) in 2021 to 33.07 per 100,000 population (95% UI: 31.35–34.79) in 2035 (Table 4; Figures 8A,B). In females, both the ASMR and ASDR of high BMI-related CRC exhibited a slightly increasing trend and gradually stabilized (Table 4; Figures 8C,D).

**Table 4:** Forecast of the global burden of colorectal cancer attributable to high body-mass index from 2022 to 2035 (per 100,000 people).

Forecast of the global burden of colorectal cancer attributable to high body mass index from 2022 to 2035 (per 100,000 people)							
Sex/Measure	2022	2023	2024	2025	2026	2027	2028
Male/ASMR	1.34 (1.32 to 1.35)	1.34 (1.32 to 1.36)	1.35 (1.32 to 1.37)	1.35 (1.32 to 1.38)	1.35 (1.32 to 1.39)	1.36 (1.32 to 1.4)	1.36 (1.32 to 1.41)
Female/ASMR	1.05 (1.03 to 1.06)	1.05 (1.03 to 1.07)	1.05 (1.02 to 1.08)	1.05 (1.02 to 1.09)	1.06 (1.01 to 1.1)	1.06 (1.01 to 1.1)	1.06 (1.01 to 1.11)
Male/ASDR	31.23 (30.94 to 31.52)	31.37 (30.9 to 31.84)	31.52 (30.89 to 32.14)	31.66 (30.91 to 32.41)	31.8 (30.93 to 32.67)	31.94 (30.96 to 32.92)	32.08 (31 to 33.16)
Female/ASDR	24.08 (23.77 to 24.39)	24.14 (23.63 to 24.65)	24.17 (23.5 to 24.83)	24.18 (23.4 to 24.96)	24.19 (23.33 to 25.06)	24.2 (23.27 to 25.13)	24.21 (23.22 to 25.19)
Sex/Measure	2029	2030	2031	2032	2033	2034	2035
Male/ASMR	1.37 (1.32 to 1.42)	1.37 (1.32 to 1.42)	1.38 (1.32 to 1.43)	1.38 (1.32 to 1.44)	1.39 (1.32 to 1.45)	1.39 (1.33 to 1.46)	1.4 (1.33 to 1.47)
Female/ASMR	1.06 (1 to 1.11)	1.06 (1 to 1.11)	1.06 (1 to 1.12)	1.06 (1 to 1.12)	1.06 (1 to 1.12)	1.06 (1 to 1.13)	1.06 (1 to 1.13)
Male/ASDR	32.22 (31.05 to 33.4)	32.36 (31.09 to 33.64)	32.51 (31.14 to 33.87)	32.65 (31.19 to 34.1)	32.79 (31.24 to 34.33)	32.93 (31.3 to 34.56)	33.07 (31.35 to 34.79)
Female/ASDR	24.21 (23.18 to 25.24)	24.22 (23.15 to 25.28)	24.22 (23.13 to 25.32)	24.23 (23.11 to 25.34)	24.23 (23.09 to 25.37)	24.23 (23.08 to 25.39)	24.24 (23.07 to 25.4)

## 4 Discussion

In recent years, due to dietary and metabolic factors, as well as unhealthy habits such as smoking and drinking, the global incidence, mortality, and DALYs rates of CRC have been increasing annually and are expected to exceed 2,200,000 new and 1,100,000 deaths by 2030 (1, 4, 26). Interestingly, a high BMI has now surpassed smoking as a major risk factor for CRC, which cannot be ignored. In this study, we systematically analyzed the global burden of CRC caused by a high BMI from 1990 to 2021, stratified by age, gender, region, and SDI. Our results revealed that there are significant differences in the burden of CRC caused by high BMI across different regions and countries, as well as among different genders and age groups. However, it is worth noting that although the number of deaths and DALYs for men and women more than doubled in 2021, after age standardization, the ASMR and ASDR for men have increased significantly over the past 32 years, while for women, they have decreased significantly. The burden of high BMI-related CRC is significantly higher in men than in women, and this gap is widening annually. This may be because women’s lifestyles are healthier compared to men, and women have much higher levels of estrogen than men. Studies have demonstrated that the expression of estrogen receptor *β* (ERβ/ESR2) is negatively correlated with the presence of colorectal polyps and tumor staging, which can reduce the risk of death in women (27, 28).

Furthermore, compared to men of the same age, women aged ≥80 years had higher mortality and DALYs related to high BMI-associated CRC. Several perspectives have supported this phenomenon. First, women tend to live longer than men do, leading to a higher proportion of elderly women in the population. Second, differences in body composition also play a role; as age increases, men tend to have a higher proportion of lean muscle mass and strength, while women have more body fat and experience faster muscle function decline compared to elderly men. These factors contribute to an increased risk of BMI-related CRC in elderly women (29, 30).

In 2021, according to the GBD 2021, there were 99,268 deaths worldwide, of which 86,308 were elderly people aged 55 years and above, accounting for nearly 87%, indicating that the burden of high BMI-related CRC is mainly concentrated in the elderly. Population growth and aging are likely to be the main causes of this phenomenon. Elderly individuals may be more susceptible to the adverse effects of high BMI, including altered glucose metabolism and insulin resistance, which increases the risk of chronic diseases (31). Furthermore, this burden is closely related to socioeconomic development and unequal distribution. While the burden of CRC caused by high BMI is higher in regions with higher SDI levels, with more than two-thirds of the deaths occurring in high SDI and high-middle SDI regions, the growth rates of ASMR and ASDR in these regions have slowed or even declined in recent years. For instance, the ASMR and ASDR in Australasia and High-income North America have significantly decreased. Contrarily, in low, low-middle, and middle SDI regions, such as South Asia and Central Sub-Saharan Africa, although the baseline burden of CRC caused by high BMI is relatively low, the number and rate of deaths and DALYs have increased significantly and may not stop growing for some time. One possible explanation is that countries with a high SDI are more likely to implement routine and early CRC screening, a mature intervention measure to reduce CRC mortality risk (32, 33). Moreover, regions with higher SDI are often more accessible to health education and awareness programs. People in these regions may be more aware of the risks of obesity and adopt healthier lifestyles and behaviors (34). In contrast, people in lower economic regions may experience greater stress due to economic difficulties and limited access to healthcare, which can lead to increased BMI and unhealthy eating habits, including high intake of energy-dense and low-nutrient foods, while reducing physical activity levels, ultimately leading to obesity (35), Consequently, countries worldwide should recognize that high BMI will eventually result in higher health burdens and take timely targeted measures, such as strengthening health education, promoting healthy weight maintenance, and encouraging active participation in physical exercise to mitigate the burden of CRC caused by high BMI.

Our research found that from 1990 to 2021, among the 21 GBD regions, although Central Europe had the highest ASMR and ASDR of high BMI-related CRC, its EAPC was much lower than that of South Asia, Southeast Asia, and East Asia. Several explanations exist for this phenomenon. First, urbanization has brought about significant lifestyle changes, with reduced physical activity, more sedentary work, and reliance on motor vehicles for transportation, leading to decreased daily activity levels. These changes increase the risk of obesity (36, 37). Second, East Asian and Southeast Asian countries are undergoing a “nutrition transition”; the deeper the transition, the higher the incidence of overweight. Traditional low-fat diets typically feature complex carbohydrates, fresh fish, meat, and leafy vegetables, which can reduce the prevalence of metabolic syndrome and increase high-density lipoprotein cholesterol levels. These have gradually shifted to modern low-nutrition, high-calorie diets, including refined starches, oils, sugars, and processed meat. This significant dietary shift has greatly increased the burden of high BMI-related CRC in these regions (38, 39).

We also found that in 1990, the higher the baseline burden of ASMR and ASDR, the lower the EAPC, exhibiting a significant negative correlation. Most countries with high baseline burdens in 1990 were developed countries, such as the United States, the United Kingdom, and Australia. They could adopt various methods, such as health education, early screening, and early intervention, to reduce the disease burden of high BMI-related CRC. Conversely, countries with low baseline burdens, such as China and India, are mostly developing countries that have experienced rapid economic development and increasingly Westernized lifestyles over the past 30 years. The increase in obesity and decrease in physical activity owing to Westernized lifestyles are important reasons for the rapid increase in CRC incidence and mortality caused by high BMI in these countries (40). In countries with a higher SDI (> 0.5) in 2021, the decline rates of ASMR and ASDR were also faster, and the EAPC revealed an inverted U-shaped relationship with SDI. In countries with a lower SDI (< 0.5), ASMR and ASDR continued to rise. The burden of BMI-related CRC varies significantly across regions and countries, and prevention and treatment strategies should be formulated based on the specific conditions in each region and country. Developing countries can take measures to help residents establish healthy lifestyles, such as actively promoting healthy diets and physical activity to avoid the increased burden of CRC caused by Westernized lifestyles. Developed countries need to take more targeted measures to reduce the prevalence of obesity and low physical activity while continuing early screening and diagnosis.

Our study has some advantages. First, our study discusses in detail the geographical and temporal trends in the global CRC burden caused by high BMI and its influencing factors, which may serve as a reference for establishing interventions to address this challenge and monitor their effectiveness. Second, our study proposes practical strategies aimed at reducing the burden on particularly affected age groups, genders, regions, and countries. Finally, our study uses the ARIMA model (a powerful time-series forecasting tool) to predict the global CRC burden related to high BMI from 2020 to 2035, enhancing our insights into future trends. However, this study has some limitations. First, the accuracy and robustness of the GBD study estimates depend on the quality and quantity of source data. Although GBD 2021 uses many strategies to improve data quality and comparability, bias is inevitable, which may affect the completeness and accuracy of the analyzed data (41). Second, BMI is an imperfect measure of obesity because individuals with high muscle mass may exhibit elevated BMI without associated health risks. An ideal measure of obesity should include waist circumference, body fat composition, and other factors, such as the muscle-to-fat ratio and metabolic health markers (34). Therefore, using BMI classification cut-off values may misestimate the impact of high BMI on CRC in specific populations. Finally, differences in the quality and quantity of data sources, etiological assessments, and diagnostic accuracy of CRC across regions may lead to heterogeneity (42).

## 5 Conclusion

In summary, current research indicates that the global burden of CRC caused by high BMI has persisted over the past 30 years and may increase in the future, particularly in countries with a lower SDI. These increasing challenges compel us to formulate targeted public health interventions and policies. Besides, according to our predictions, global ASMR and ASDR may exhibit an upward trend between 2022 and 2035. We hope that our study can provide policymakers with insights into formulating disease control and prevention strategies tailored to the specific conditions of different countries, particularly those with relatively low SDI, according to their socioeconomic conditions.

## Data Availability

Publicly available datasets were analyzed in this study. This data can be found at: http://ghdx.healthdata.org/gbd-results-tool.

## Footnotes

^1^http://ghdx.healthdata.org/gbd-results-tool

## Supplementary material

SUPPLEMENTARY FIGURE S1 Population attributable fraction of colorectal cancer deaths attributable to high BMI by 5 SDI areas and 21 GBD regions in 1990 and 2021 (A); Population attributable fraction of colorectal cancer DALYs attributable to high BMI by 5 SDI areas and 21 GBD regions in 1990 and 2021 (B); BMI, body-mass index; SDI, sociodemographic index; DALYs, disability-adjusted life-years.
