# Supplementary figures and images for: The global burden of colorectal cancer attributable to high body-mass index in 204 countries and territories: findings from 1990 to 2021 and predictions to 2035
Source: Front Nutr. 2024 Nov 20;11:1473851. doi: 10.3389/fnut.2024.1473851 (PMC11614609; doi:10.3389/fnut.2024.1473851)

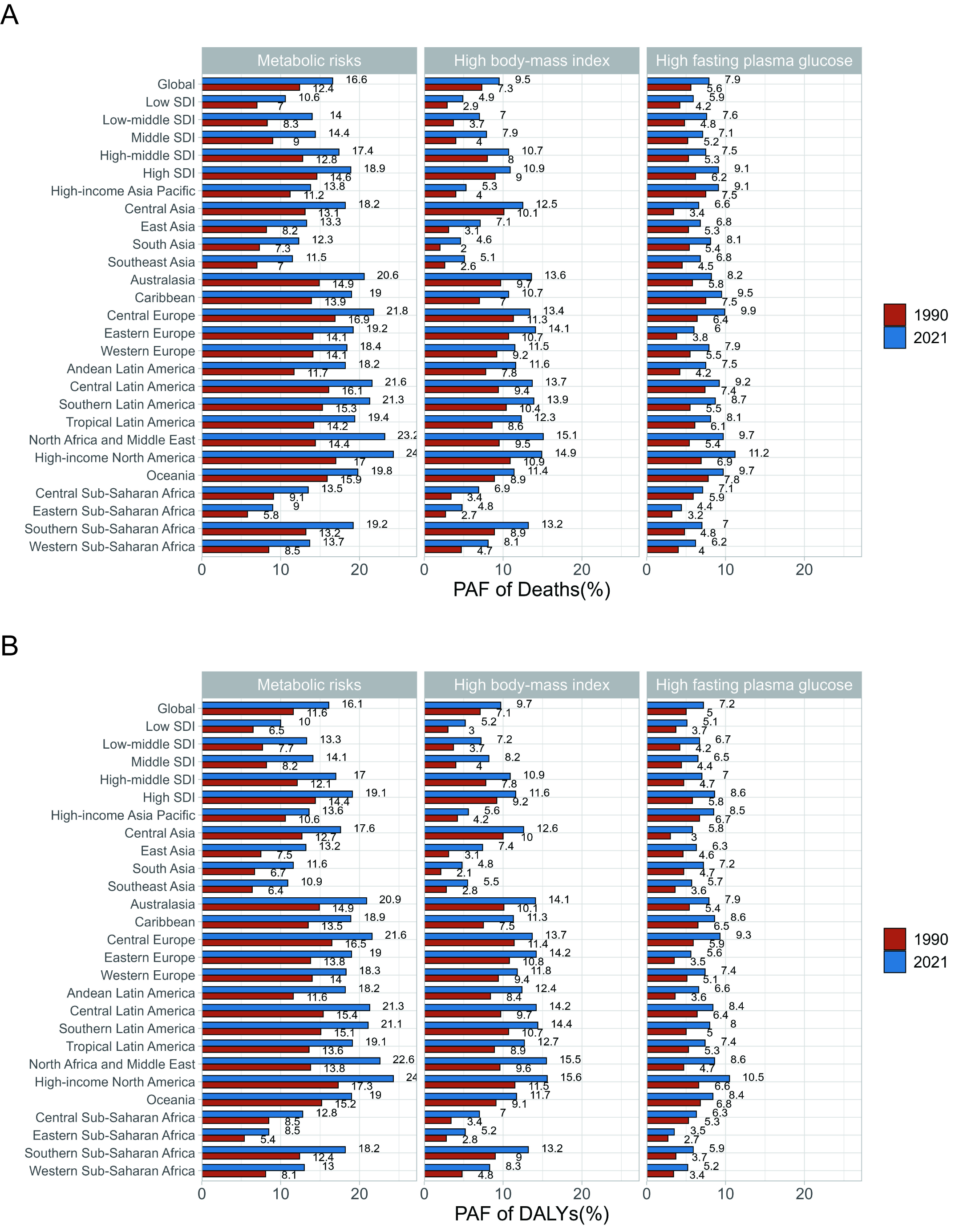

Supplement: SUPPLEMENTARY FIGURE S1 — Population attributable fraction of colorectal cancer deaths attributable to high BMI by 5 SDI areas and 21 GBD regions in 1990 and 2021 (A); Population attributable fraction of colorectal cancer DALYs attributable to high BMI by 5 SDI areas and 21 GBD regions in 1990 and 2021 (B); BMI, body-mass index; SDI, sociodemographic index; DALYs, disability-adjusted life-years. [file Image_1.TIF]
